# Primary Health Care, work and stress of nursing professionals: a synthesis of evidence

**DOI:** 10.1590/0034-7167-2025-0562

**Published:** 2026-09-28

**Authors:** Lusicleide Galindo da Silva Moraes, Ana Claudia Morais Godoy Figueiredo, Maria Lúcia Silva Servo

**Affiliations:** I Universidade Estadual de Feira de Santana. Feira de Santana, Bahia, Brazil; II Fundação de Ensino e Pesquisa em Ciências da Saúde. Brasília, Distrito Federal, Brazil

**Keywords:** Nurses, Work, Psychological Stress, Occupational Stress, Primary Health Care., Enfermeras, Trabajo, Estrés Psicológico, Estrés Ocupacional, Atención Primaria de Salud.

## Abstract

**Objectives:**

to map the characteristics of work-related stress among nurses working in Primary Health Care.

**Methods:**

a scoping review, conducted in 2024, with evidence tracking in six health databases, namely Embase, PubMed, Scopus, Web of Science, LILACS, and SciELO.

**Results:**

a total of 13 selected articles showed that the assessed outcome is related to psychological, emotional, and personal issues. However, it was more evident when related to attributes of work, organization, management, and the system.

**Conclusions:**

studies indicate that the stress experienced by nurses in Primary Health Care was characterized by poor working conditions and work overload, lack of professional qualifications, interpersonal conflicts, high psychological demands, high professional requirements, lack of social support, and scarcity of human, financial, and material resources.

## RESUMEN

**Objetivos:**

mapear las características relacionadas con las condiciones laborales y de estrés del personal de enfermería que trabaja en Atención Primaria de Salud.

**Métodos:**

una revisión de alcance, realizada en 2024, rastreó la evidencia en seis bases de datos relacionadas con la salud: Embase, PubMed, Scopus, *Web of Science*, LILACS y SciELO.

**Resultados:**

un total de 13 artículos seleccionados mostraron que el resultado evaluado se relaciona con aspectos psicológicos, emocionales y personales. Sin embargo, fue más evidente al relacionarse con los atributos del trabajo, la organización, la gestión y el sistema.

**Conclusiones:**

los estudios indican que el estrés experimentado por las enfermeras en Atención Primaria de Salud se caracteriza por malas condiciones de trabajo y sobrecarga de trabajo, falta de cualificación profesional, conflictos interpersonales, altas exigencias psicológicas, altas exigencias profesionales, falta de apoyo social y escasez de recursos humanos, financieros y materiales.

## INTRODUCTION

Access to healthcare services should primarily occur through Primary Health Care (PHC). The Health Care Network has the Family Health Strategy as its main structuring axis^(1)^. PHC consists of developing actions in an individual and/or group context aimed at promoting and protecting health, as well as preventing diseases and health problems, considering the unique characteristics of individuals and their community^(2)^.

In this context, the nurses’ role in PHC emerges; they are generally the first professionals to welcome citizens to the health unit, carrying out their activities in a systematic and scientific manner. Through care provision to users, nurses seek to meet their needs, considering the territory’s epidemiological characteristics^(2)^. Thus, PHC nurses’ work is conducted through the development of care and management activities, facilitating the relationship between the population’s needs, the team workforce, and the organizational environment^(3)^.

However, the difficulties faced in performing their duties cause stress, which can compromise their health due to feelings of inadequacy^(4)^. In this context, negative experiences in the workplace raise stress levels, generating emotional overload and undermining the quality of healthcare provided to the population^(5)^. In addition to this, there is exposure to multiple factors of physical, chemical, biological, mental, and social origin, which lead to psychological stress and illness^(6)^.

Psychological stress presents itself as a set of manifestations in the body with the potential to cause illness due to the difficulty or impossibility of carrying out the activities and meeting the work environment demands. Thus, stress among PHC nurses can be caused by excessive demands, leading to mental overload, such as time constraints, speed, intensity, and less control over work activities due to decision-making and the use of intellectual skills^(4)^.

Given the above, it is understood that the psychological stress encountered in PHC nurses’ work needs to be studied and discussed so that the main stressors and gaps in scientific knowledge regarding evidence, focusing exclusively on the subject in question, can be identified. Furthermore, conducting a scoping review allows for the identification of existing knowledge and underexplored areas, in order to guide future research and strengthen public policies and management practices aimed at valuing and protecting PHC nurses’ work.

## OBJECTIVES

To map the characteristics of work-related stress among nurses working in Primary Health Care.

## METHODS

### Ethical aspects

Since this is a scoping review-type evidence synthesis that consulted records available on the internet, there was no need to submit the proposal to a Research Ethics Committee.

### Study design

This review is a scoping review, providing a broad overview of evidence, and can also be used to clarify questions about the nature and diversity of existing evidence and/or knowledge^(7)^.

For the study, JBI and Preferred Reporting Items for Systematic Reviews and Meta-Analyses extension for Scoping Reviews (PRISMA-ScR) checklist recommendations were followed^(7)^.

### Research question identification

The search for evidence was conducted by structuring the strategy using the acronym PCC: Population (Nurses); Concept (Stress); and Context (PHC). The research question was: can working in PHC trigger stress in nurses working in this service?

### Relevant study identification

Two researchers conducted a search for evidence in March 2024 using the Coordination for the Improvement of Higher Education Personnel’s Journals Portal, in the following databases: two specific to the health field (Embase and U.S. Medical Literature Analysis and Retrieval System Online via PubMed); two multidisciplinary databases (Scopus and Web of Science); two regional databases (LILACS and SciELO via Virtual Health Library); and one grey literature source (ProQuest via Web of Science).

Following standardized concepts for information representation and assisting in the search for information in online libraries^(8)^, descriptors were tracked in a controlled vocabulary of indexed terms, such as Medical Subject Headings (MeSH) and Emtree, as well as free and similar terms. Initially, tracking was carried out by cross-referencing MeSH terms, except for the Embase database: Nurses AND Work AND Stress AND Primary Health Care. The following MeSH descriptors were used to search for evidence: Nurses AND Work AND Psychological Stress AND Occupational Stress AND Primary Health Care. For Embase, the specific Emtree descriptors were used: ‘’Community Health Nurse’’ AND ‘’Mental Stresses’’ AND ‘’Health Care, Primary’’. Furthermore, the studies’ reference lists tracked to investigate more studies related to the topic in question. The search strategy’s structure is previously available in a protocol published on the Open Science Framework^(9)^.

Subsequently, a preliminary search was conducted to determine if retrieved studies were being captured and also to identify new unindexed terms. Subsequently, unindexed terms were detected. A new search strategy was constructed and, for its qualification, a sensitivity analysis was performed by another evaluator using the Peer Review of Electronic Search Strategies. Complementarily, the studies’ reference lists were tracked to investigate more studies related to the topic in question.

### Study selection

In the searches carried out in databases, 8,666 records were identified, as shown in Figure 1. Of these, 1,110 were removed because they were duplicates. Thus, 7,556 were selected for screening, leaving 35 studies for the full-text phase, with 22 excluded and 13 selected for review (Figure 1).

**Figure 1:**
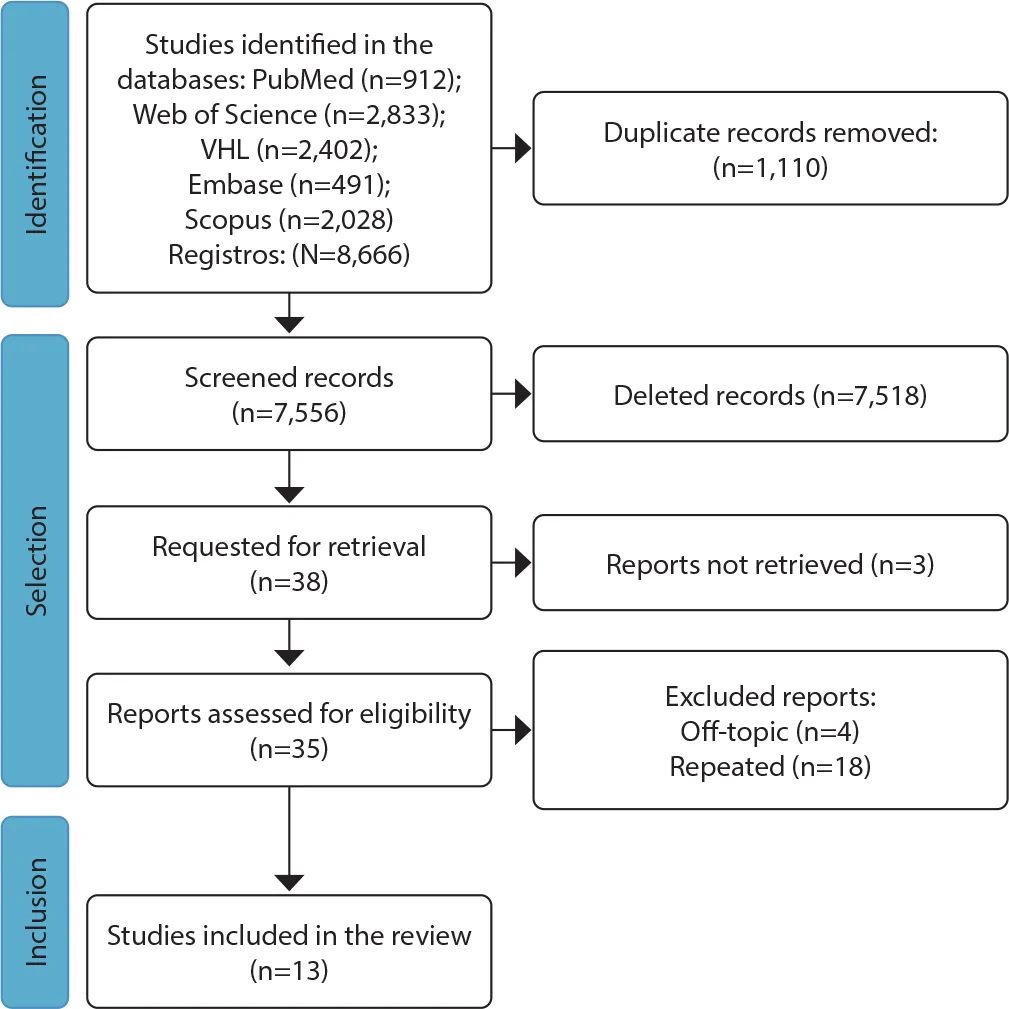
Study selection flowchart

Studies in any language, without time restrictions, available online in full text, that addressed stress in nurses working in PHC were included in the review. Studies addressing the following were excluded: post-traumatic stress, as it deals with a psychopathology associated with intense traumatic situations, such as life-threatening situations and catastrophes, events that are not part of the daily routine of the studied context; burnout syndrome or professional exhaustion syndrome, to avoid conceptual overlap, which could compromise the analysis, since the phenomenon requires its own diagnostic instruments and criteria that differ from psychological stress; coping strategies, as the study seeks the characteristics of the stress experienced and not how nurses deal with it; stress from a physiological perspective, as it seeks to understand stress from a subjective perspective based on the perception of work-related experiences and not from a biological perspective; quality of life and those who approach mindfulness meditation, because it does not directly address stress.

Record selection and initial assessment, conducted in two stages, began with the screening of titles and abstracts, then full text, according to established eligibility criteria.

In the first phase, the Rayyan - Intelligent Systematic Review software was used^(10)^. The process was carried out by two researchers, and blinding was maintained until selection was finalized.

Full-text assessment comprised the second phase and was carried out by reading the files in PDF format. Upon completion of each phase, blinding was removed, and conflicts were consensually discussed, analyzed, and resolved by the researchers.

Subsequently, a flowchart was created (Figure 1). This summarized the search and selection process for the studies in this review, showing the quantity from each database, as well as the studies that were included and excluded, in addition to the total selected for assessment and synthesis (Figure 1).

### Data analysis

The methodological quality of the articles selected for the scoping review was assessed using the JBI Critical Appraisal Tools to verify article reliability, relevance, and results, considering cross-sectional analytical studies and studies that reveal prevalence data^(11)^.

To assess cross-sectional analytical studies, methodological aspects were considered, such as sample selection, research participant and environment description, exposure and outcome measurement, confounding factors, and appropriate statistical analysis. For studies revealing prevalence data, the target population, study participants, sample size, participant and environment description, data analysis coverage according to the sample, methods used for exposure measurement, and statistical analysis were verified.

The studies were analyzed independently by each researcher, and data were extracted and compiled as follows: the first addresses the studies’ main characteristics; and the second relates to the approach to work-related stress.

The extracted information was organized into a spreadsheet created using Microsoft Office Excel^®^, in which the results were described. A descriptive summary of results was presented in tables to facilitate visualization and understanding of the data.

For data presentation, the recommendations of international PRISMA-ScR guidelines were considered, and data were discussed in accordance with relevant literature^(7)^.

## RESULTS

For better presentation of results, the data obtained were organized into two charts, according to the categories: Studies that make up the sample (Chart 1); and Stress approach (Chart 2).

**Chart 1:** Studies that make up the sample

ID code	Title	Country/year	Study design
1^(12)^	Work-related stress among nursing staff working in government hospitals and primary health care centres	Saudi Arabia 2018	Cross-sectional study
2^(13)^	Do nurses feel stressed? A perspective from primary health care	Lithuania 2013	Descriptive study
3^(14)^	Estrés laboral en profesionales de la atención primaria durante la pandemia de COVID-19: estudio de métodos mixtos	Brazil 2023	Exploratory study
4^(15)^	Estrés percibido en trabajadores de atención primaria	Chile 2019	Cross-sectional study
5^(16)^	Identification of Causes of the Occupational Stress for Health Providers at Different Levels of Health Care	Bosnia and Herzegovina 2017	Cross-sectional study
6^(17)^	Increased Organizational Stress in Primary Care: Understanding the Impact of the COVID-19 Pandemic, Medicaid Expansion, and Practice Ownership	United States 2023	Mixed-methods study: quantitative and qualitative
7^(18)^	Nurses’ cognitive structural models of work-based stress	United Kingdom 1999	Descriptive study
8^(19)^	Perceived job stress among community nurses: A multi-center cross-sectional study	China 2018	Cross-sectional study
9^(20)^	Predictors of work-related stress among nurses working in primary and secondary health care levels in Dammam, Eastern Saudi Arabia	Saudi Arabia 2014	Cross-sectional study
10^(21)^	Prevalence and associated factors of stress among primary health care nurses in Saudi Arabia, a multi-center study	Saudi Arabia 2021	Cross-sectional study
11^(22)^	Stress and turnover intention among healthcare workers in Saudi Arabia during the time of COVID-19: Can social support play a role?	Saudi Arabia 2021	Cross-sectional study
12^(23)^	The association of primary healthcare nurses’ perceived stress with organizational culture and climate in a team context	Lithuania 2019	Cross-sectional study
13^(24)^	Job stress among general practitioners and nurses in primary care in England	England 1999	Descriptive study

**Chart 2:** Stress management

Stress management		
ID code	Stress characterization	Study applicability
1^(12)^	Work overload, lack of professional qualifications, interpersonal conflicts, age, gender, marital status, and nationality were the factors that contributed to stress.	Continuing education programs for nurses to cope with stress should be designed according to the type of workplace. Public policies should focus on establishing a healthy work environment, free from stress, understaffing, and excessive workload.
2^(13)^	Stress was linked to high psychological demands and a high degree of professional expectation.	The negative impacts on workers’ mental health need to be mitigated through strategies that promote health, quality of life, and social support in the primary healthcare workplace.
3^(14)^	It considered psychosocial aspects related to work, based on the demand-control and social support model postulated by Karasek and Theorell.	Employers can invest in improving working conditions, and national bodies can emphasize monitoring and supervision in order to reduce the percentage of workers affected by illnesses caused by mental stress.
4^(15)^	An approach to perceived stress and its individual, professional, and group aspects, including psychosocial and environmental risks related to work.	The study focuses on cost-effective actions to provide better worker health conditions aimed at minimizing work-related problems that may cause illness, as well as promoting physical, mental, and social health.
5^(16)^	Addressing organizational stress.	The need for work organization, continuing education promotion, structural improvement, and planning are essential elements for minimizing organizational work stressors.
6^(17)^	Overburdening, resource scarcity, and incomplete staffing are factors that contribute to stress among primary care workers.	Effective strategies from healthcare systems are fundamental and should be adopted with a view to investing in professionals, providing adequate resources to expand services, and reducing administrative bureaucracy.
7^(18)^	Stress is approached from the perspective of individuals’ conceptions of what causes stress.	This study addresses the ongoing need to develop a solid understanding of the main causes of work-related stress and their interrelationships, since the consequences of a stressed and demoralized nursing workforce interfere with the quality of patient care and the risk profile.
8^(19)^	Stress is related to an individual’s perception of the demands placed upon them and their ability to meet those demands.	Managers should intervene through programs that promote on-the-job training, aiming to adopt stress-relief measures to balance work and family life, reduce work shifts, and support career development, wages, and benefits.
9^(20)^	Work-related stress is caused or exacerbated by work. It is a substantial and growing concern for workers, advocates, employers, occupational health and safety regulators, and workers’ compensation programs.	An appropriate strategy is recommended within healthcare organizations to investigate stress management in healthcare settings. Furthermore, intervention programs should be developed to identify and alleviate the sources and effects of stress, including increased training, support, and improved working conditions.
10^(21)^	Occupational stress was classified as a negatively perceived feeling, due to the inability to cope with organizational work demands.	A comprehensive interventional approach is needed to minimize and prevent stress among nurses. Organizational interventions should be aimed at reducing stress and mitigating the impact of workplace stressors, including workload and stress management training programs.
11^(22)^	Social support was pointed out as a strategy to minimize the impact of stress.	Social support can be used as a coping strategy to reduce stress and turnover among healthcare professionals.
12^(23)^	Poor patient care, poor communication, and lack of support were identified as factors that prevent nurses from using their full potential to provide the highest quality care to their patients, and this contributes to stress.	Further studies are recommended to establish the impact of the relationship between organizational culture and climate on stress.
13^(24)^	Job satisfaction and mental well-being were considered when addressing stress.	There has been little work done on the effects of mental health on patient care and its consequences, which reinforces the idea that this is an area that requires more research.

For study characterization, the article identification code, country/year, and study design were considered, as presented in Chart 1.

For the approach to stress, aspects related to the article were considered through the identification code, stress characterization, and study applicability (Chart 2).

## DISCUSSION

According to the studies that make up the sample, presented in Chart 1, it was found that the titles presented are related to work stress in the context of PHC. The publication period was from 1999 to 2023, with four in Saudi Arabia, two in Lithuania, one in Brazil, one in Chile, one in Bosnia and Herzegovina, one in the United States, one in the United Kingdom, one in China, and one in England. Concerning study design, eight cross-sectional studies, three descriptive studies, one exploratory study, and one mixed-methods study (qualitative and quantitative) were identified^(12-24)^.

In relation to the reviews found, they all addressed stress in general terms^(25-48)^. There was difficulty in finding reviews that assessed only the stress experienced by nurses at work in PHC. Therefore, there was a concern to select studies considering the similarity between healthcare services and the nurses’ role, finding that stress is present in the target population investigated.

From this perspective, an integrative review article showed that the prevalence of stress among PHC workers was classified as low to moderate, being associated with conflicts between team members, mainly physicians and nurses. Furthermore, it is highlighted that the various interfaces of the workday in the workplace and at home, as well as family care, make nurses more prone to stress^(43)^.

Regarding the approach to stress (Chart 2), it was characterized by work overload, lack of professional qualifications, and interpersonal conflicts, including sociodemographic aspects that favor psychological stress, such as age, sex, marital status, and nationality. Furthermore, stress was related to high psychological demands, a high degree of professional expectation, and a lack of social support^(12-24)^.

In addition, evidence indicates that low social support in the workplace is associated with social and psychological aspects, such as occupational stress. On the other hand, high social support contributes to protection against the deleterious effects on mental health, favoring work capacity. Therefore, it is recommended that interventions and implementation of social support be carried out in a humanized, safe and professionally recognized manner, focusing on reducing workload and the harmful effects of the work environment^(44)^.

Regarding study applicability (Chart 2), among the measures indicated to minimize the impacts of stress at work, continuing education programs directed at the workplace and public policies on worker health in the context of PHC stood out as strategies to have a safe, welcoming workspace, free from stress, staff shortages, and a high workload^(12-24)^.

However, the lack of development of actions aimed at promoting health, quality of life, and social support was emphasized. Investments in improving working conditions, as well as oversight and supervision by national agencies, were presented as measures that can prevent or reduce the occurrence of occupational diseases or injuries. As for organizational structure and planning, when well-structured, they were presented as essential elements to minimize risks and, consequently, psychological stress^(12-24)^.

These findings demonstrate that, within the scope of PHC, care and monitoring of nurses’ physical and mental health are essential, considering the stressors and occupational risks present in the work environment. Reorganizing the work process, using health technologies appropriately, and adopting preventive measures such as improving working conditions, conflict management, ongoing team education activities, breaks during the workday, professional development, development of humanization policies, psychological support, and support for occupational stress are strategies that should be adopted to make the workday less exhausting and more productive, with less stress and illness^(45)^.

In light of this, the studies analyzed also showed that better working conditions require organizational and individual interventions, involving investment in professionals, appropriate application of resources to expand services, reduction of administrative bureaucracy, adoption of stress relief measures, reduction of work shifts, reduction of employee turnover, support for career development, wages and benefits, stress management training, and coping programs^(12-24)^.

In line with these findings, a review study reinforced that the precarious working conditions of nurses are aggravated by a scarcity of material resources, a shortage of professionals, precarious employment contracts, and low wages. This scenario contributes to the overload of these professionals, who are also exposed to physical, chemical, biological, and ergonomic risks, making them vulnerable to the development of physical and mental illnesses, interfering with their quality of life and the care provided. The competence required of nurses and the imbalance between demands and working conditions cause exhaustion, stress, and psychological and physiological damage^(46)^.

However, it was found that creating healthy work environments in PHC, free from stress and stressors, poses a challenge for managers and workers who envision strategies to address the various situations that limit and sometimes prevent the adoption of individual and collective protection measures. Therefore, the process of changing this reality is complex, requiring mobilization and dialogue among actors, and a struggle to consolidate public policies for worker health^(48)^.

Supporting this theme, an integrative review article pointed out that the qualification of PHC workers is an essential component for improving the care provided in health^(47)^. Therefore, it becomes essential to understand and accommodate the demands for professional updating and improvement, since PHC aims to guide policies for strengthening health systems, welcoming its users in a comprehensive, effective, and efficient manner.

### Study limitations

The limitations of this scoping review include the scarcity of studies addressing stress among nurses working in PHC, especially in Brazil. This context demands more studies that explore the topic and contribute to filling existing gaps in the literature.

Furthermore, study heterogeneity partially compromises the direct answer to the research question, and may interfere with the interpretation and generalization of the review findings.

### Contributions to nursing, health, or public policy

This scoping review identified the source of stress among PHC nurses, demonstrating the urgent need to reorganize PHC services, including health budget reform, to ensure feasible health planning with improved working conditions, professional recognition, autonomy, and respect.

Based on these results, and the identification of contributing factors to stress, it is suggested that measures can be adopted by professionals on-site, such as: dividing tasks and responsibilities among all team members; conducting a reconnaissance of the work area; holding meetings with community leaders and representatives of the assigned population to understand their needs and thus plan and implement actions to meet them, directing available resources more efficiently and effectively; and offering social and psychological support to PHC nurses.

## CONCLUSIONS

Studies have indicated that the psychological stress experienced by PHC nurses stems from exposure to stressors in the workplace, and the main causes are related to working conditions, organization, and management. Thus, psychological stress is characterized by poor working conditions and work overload, lack of professional qualifications, interpersonal conflicts, high psychological demands, a high degree of professional expectation, lack of social support, as well as a lack of human, financial, and material resources.

The study also found that individual and professional factors, family issues, the ability to meet job demands, and the capacity to cope with organizational demands all contributed to the influence of stressors present in the work environment.

It is recommended to pay attention to the need for reorganizing PHC nurses’ work, aiming at professional qualification, improvement of working conditions, identification of stressors, elimination of occupational risks, adequate infrastructure, and sufficient human, material and financial resources to meet the demands.

Given the above, the studies emphasized measures aimed at preventing or reducing psychological stress among PHC nurses, including continuing education programs and a healthy work environment with a view to promoting health, quality of life and well-being.

## Data Availability

The research data are available within the article.
