# Supplementary material for: Primary Health Care, work and stress of nursing professionals: a synthesis of evidence
Source: Rev Bras Enferm. 2026 Sep 28;79:e20250562. doi: 10.1590/0034-7167-2025-0562 (PMC13623384; doi:10.1590/0034-7167-2025-0562)
Supplement: Translation

# Translation: Atenção Primária à Saúde, trabalho e estresse das profissionais enfermeiras: uma síntese de evidências

**DOI:** 10.1590/0034-7167-2025-0562pt

**Authors:** Lusicleide Galindo da Silva Moraes, Ana Claudia Morais Godoy Figueiredo, Maria Lúcia Silva Servo

**Affiliations:** I Universidade Estadual de Feira de Santana. Feira de Santana, Bahia, Brasil; II Fundação de Ensino e Pesquisa em Ciências da Saúde. Brasília, Distrito Federal, Brasil

**Keywords:** Enfermeiras, Trabalho, Estresse Psicológico, Estresse Ocupacional, Atenção Primária à, Saúde.

## Abstract

**Objetivos:**

mapear as características relacionadas ao trabalho e à condição de estresse de enfermeiras que atuam na Atenção Primária à Saúde.

**Métodos:**

revisão de escopo, realizada em 2024, com rastreamento de evidências em seis bases de dados da área da saúde, a saber Embase, PubMed, Scopus, *Web of Science*, LILACS e SciELO.

**Resultados:**

um total de 13 artigos selecionados mostrou que o desfecho avaliado está relacionado a questões psicológicas, emocionais e pessoais. Todavia, mostrou-se mais evidente quando relacionado a atributos do trabalho, da organização, da gestão e do sistema.

**Conclusões:**

os estudos apontam que o estresse apresentado pelas enfermeiras na Atenção Primária à Saúde foi caracterizado pelas más condições e sobrecarga de trabalho, déficit na qualificação profissional, conflitos interpessoais, elevada demanda psicológica, alto grau de exigência profissional, falta de apoio social, e escassez de recursos humanos, financeiros e materiais.

## INTRODUÇÃO

O acesso da população aos serviços de saúde deve ocorrer preferencialmente pela Atenção Primária à Saúde (APS). A Rede de Atenção à Saúde tem a Estratégia Saúde da Família como o seu principal eixo estruturante^(1)^. A APS consiste em desenvolver ações em um contexto individual e/ou em grupo que visam à promoção e proteção da saúde, além de prevenção de doenças e agravos, considerando as singularidades da pessoa e do seu coletivo^(2)^.

Nesse contexto, emerge o papel da enfermeira na APS, aquela que, geralmente, é a primeira profissional a acolher o cidadão na unidade de saúde, exercendo as suas atividades de forma sistemática e científica. Por meio da oferta do cuidado aos usuários, a enfermeira busca atender às necessidades considerando as características epidemiológicas do território^(2)^. Assim, o trabalho das enfermeiras na APS é conduzido através do desenvolvimento de atividades assistenciais e gerenciais, facilitando a relação entre as necessidades da população, a força de trabalho da equipe e o ambiente organizacional^(3)^.

No entanto, as dificuldades enfrentadas no desempenho das suas atividades ocasionam o estresse, podendo comprometer a saúde pelo sentimento de incapacidade^(4)^. Nesse contexto, as experiências negativas vivenciadas no local de trabalho elevam os níveis de estresse, gerando sobrecarga emocional e desqualificando o cuidado à saúde dispensado à população^(5)^. Associado a isso, existe a exposição a múltiplos fatores de origem física, química, biológica, mental e social, que levam ao estresse psicológico e ao adoecimento^(6)^.

O estresse psicológico se apresenta com um conjunto de manifestações no organismo com potencial de causar o adoecimento devido à dificuldade ou impossibilidade de desenvolver as atividades e as demandas existentes no ambiente laboral. Assim, o estresse das enfermeiras na APS pode ser causado pelo excesso de demandas, que ocasionam sobrecarga mental, como tempo, velocidade, intensidade e menos controle das atividades laborais devido à tomada de decisão e ao uso das habilidades intelectuais^(4)^.

Diante do exposto, compreende-se que o estresse psicológico encontrado no trabalho das enfermeiras na APS precisa ser estudado e discutido para que os principais estressores e as lacunas do conhecimento científico acerca das evidências, com enfoque exclusivo na temática em questão, sejam identificados. Além disso, a realização da revisão de escopo permite identificar o conhecimento existente e as áreas pouco exploradas, a fim de orientar pesquisas futuras e fortalecer políticas públicas e práticas de gestão voltadas à valorização e proteção do trabalho das enfermeiras na APS.

## OBJETIVOS

Mapear as características relacionadas ao trabalho e à condição de estresse de enfermeiras que atuam na Atenção Primária à Saúde

## MÉTODOS

### Aspectos éticos

Por se tratar de uma síntese de evidências do tipo revisão de escopo, que consultou registros disponíveis na internet, não houve necessidade de submeter a proposta ao Comitê de Ética em Pesquisa.

### Desenho do estudo

A revisão é do tipo revisão de escopo, fornecendo uma visão geral e ampla das evidências, podendo também ser utilizada para elucidar questões sobre a natureza e a diversidade das evidências e/ou conhecimentos existentes^(7)^.

Para a realização do estudo, foram seguidas as recomendações do JBI e do *checklist Preferred Reporting Items for Systematic Reviews and Meta-Analyses extension for Scoping Reviews* (PRISMA-ScR)^(7)^.

### Identificação da questão de pesquisa

A busca de evidências ocorreu por meio da estruturação da estratégia considerando o acrônimo PCC: População (Enfermeiras); Conceito (Estresse); e Contexto (APS). A pergunta de pesquisa foi: o trabalho na APS pode desencadear estresse nas enfermeiras atuantes neste serviço?

### Identificação dos estudos relevantes

Duas pesquisadoras conduziram a busca de evidências, em março de 2024, por meio do Portal de Periódicos da Coordenação de Aperfeiçoamento de Pessoal de Nível Superior, nas seguintes bases de dados: duas específicas da área da saúde (Embase e *U.S. Medical Literature Analysis and Retrieval System Online* via PubMed); duas multidisciplinares (*Scopus* e *Web of Science*); duas bases regionais (LILACS e SciELO via Biblioteca Virtual em Saúde); e uma fonte da literatura cinzenta (*ProQuest* via *Web of Science*).

Seguindo conceitos padronizados para a representação da informação e auxiliando na busca por informações em bibliotecas *online* ^(8)^, os descritores foram rastreados em um vocabulário controlado de termos indexados, como *Medical Subject Headings* (MeSH) e Emtree, além de termos livres e similares. Inicialmente, foi realizado o rastreio por meio do cruzamento dos termos MeSH, exceto para a base Embase: *Nurses AND Work AND Stress AND Primary Health Care.* Os descritores MeSH a seguir foram usados para busca de evidências: *Nurses AND Work AND Psychological Stress AND Occupational Stress AND Primary Health Care.* Para a Embase, utilizaram-se os descritores específicos Emtree: *‘’Community Health Nurse’’ AND ‘’Mental Stresses’’ AND ‘’Health Care, Primary’’*. Além disso, houve o rastreio das listas de referências dos estudos para investigar mais estudos relacionados com a temática em questão. A estruturação da estratégia de busca está disponível previamente em protocolo publicado no *Open Science Framework* ^(9)^.

Em seguida, foi realizada uma busca preliminar para detectar se os estudos recuperados estavam sendo captados e também identificar novos termos não indexados. Na sequência, foram detectados os termos não indexados. Foi construída uma nova estratégia de busca e, para sua qualificação, foi realizada a análise de sensibilidade por outro avaliador por meio do *Peer Review of Electronic Search Strategies.* De forma complementar, houve o rastreio das listas de referências dos estudos para investigar mais estudos relacionados à temática em questão.

### Seleção dos estudos

Nas buscas realizadas em bases de dados, foram identificados 8.666 registros, conforme Figura 1. Destes, foram removidos 1.110, por se tratar de duplicatas. Assim, foram selecionados para triagem 7.556, restando ao final 35 estudos para a fase de texto completo, com 22 foram excluídos e 13 selecionados para a revisão (Figura 1).

**Figura 1:** Fluxograma de seleção de estudos

Foram incluídas na revisão publicações em quaisquer idiomas, sem recorte temporal, disponíveis *online,* em texto completo, que abordassem o estresse em enfermeiras que atuavam na APS. Foram excluídos estudos que abordavam: estresse pós-traumático, por tratar de uma psicopatologia associada a situações traumáticas intensas, como ameaça à vida e catástrofes, eventos que não fazem parte do cotidiano do contexto estudado; síndrome de *burnout* ou síndrome do esgotamento profissional, para evitar sobreposição conceitual, o que poderia comprometer a análise, pois o fenômeno exige instrumentos diagnósticos e critérios próprios que se diferenciam do estresse psicológico; estratégias de *coping*, pois o estudo busca as características do estresse vivenciado e não a forma como as enfermeiras lidam com ele; estresse na perspectiva fisiológica, por buscar compreender o estresse na perspectiva subjetiva a partir da percepção das experiências relacionadas ao trabalho e não de forma biológica; qualidade de vida e aqueles que abordam meditação *mindfullness* (atenção plena), por não tratar diretamente do estresse.

A seleção e a avaliação inicial dos registros, conduzidas em duas etapas, iniciaram pelo rastreamento de títulos e resumos, depois texto completo, de acordo com os critérios de elegibilidade estabelecidos.

Na primeira fase, utilizou-se o *software* Rayyan - *Intelligent Systematic Review* ^(10)^. O processo foi realizado por duas pesquisadoras, e o cegamento foi mantido até finalizar a seleção.

A avaliação do texto completo compôs a segunda fase, e foi realizada através da leitura dos arquivos no formato PDF. Ao concluir cada fase, o cegamento foi retirado, e os conflitos foram consensualmente discutidos, analisados e resolvidos pelas pesquisadoras.

Posteriormente, foi elaborado um fluxograma (Figura 1). Assim, foi sintetizando o processo de busca e seleção dos estudos desta revisão, mostrando a quantidade de cada base de dados, bem como os estudos que foram incluídos e excluídos, além do total selecionado para avaliação e síntese (Figura 1).

### Análise dos dados

A avaliação da qualidade metodológica dos artigos selecionados para a revisão de escopo foi executada através da *Critical Appraisal Tools* do JBI para averiguar a confiabilidade, relevância e resultados dos artigos, considerando os estudos analíticos transversais e estudos que revelam dados de prevalência^(11)^.

Para avaliação dos estudos analíticos transversais, foram considerados aspectos metodológicos, como a seleção da amostra, descrição dos participantes da pesquisa e do ambiente, medida da exposição e desfecho, fatores de confusão, e análise estatística apropriada. Para os estudos que revelam dados de prevalência, verificaram-se a população-alvo, participantes do estudo, tamanho da amostra, descrição dos participantes e do ambiente, cobertura da análise de dados conforme a amostra, métodos utilizados para medida de exposição, e análise estatística.

Os estudos foram analisados por cada pesquisadora, de forma independente, e os dados foram extraídos e compilados da seguinte forma: a primeira aborda as principais características dos estudos; e a segunda é relacionada à abordagem do estresse relacionado ao trabalho.

As informações extraídas foram organizadas em uma planilha construída com auxílio do *software Microsoft Office Excel^®^*, no qual os resultados foram descritos. A síntese descritiva dos resultados encontrados foi apresentada em quadros, de forma a favorecer a visualização e compreensão dos dados.

Para apresentação dos dados, foram consideradas as recomendações do guia internacional PRISMA-ScR, e os dados foram discutidos de acordo com a literatura pertinente^(7)^.

## RESULTADOS

Para melhor apresentação dos resultados, os dados obtidos foram organizados em dois quadros, de acordo com as categorias: Estudos que compõem a amostra (Quadro 1); e Abordagem do estresse (Quadro 2).

**Quadro 1:** Estudos que compõem a amostra

Código ID	Título	País/ano	Tipo de estudo
1^(12)^	*Work-related stress among nursing staff working in government hospitals and primary health care centres*	Arábia Saudita 2018	Estudo transversal
2^(13)^	*Do nurses feel stressed? A perspective from primary health care*	Lituânia 2013	Estudo descritivo
3^(14)^	*Estrés laboral en profesionales de la atención primaria durante la* *pandemia de COVID-19: estudio de métodos mixtos*	Brasil 2023	Estudo exploratório
4^(15)^	*Estrés percibido en trabajadores de atención primaria*	Chile 2019	Estudo transversal
5^(16)^	*Identification of Causes of the Occupational Stress for Health Providers at Different Levels of Health Care*	Bósnia e Herzegovina 2017	Estudo transversal
6^(17)^	*Increased Organizational Stress in Primary Care: Understanding the Impact of the COVID-19 Pandemic, Medicaid Expansion, and Practice Ownership*	Estados Unidos 2023	Estudo misto: quantitativo e qualitativo
7^(18)^	*Nurses’ cognitive structural models of work-based stress*	Reino Unido 1999	Estudo descritivo
8^(19)^	*Perceived job stress among community nurses: A multi-center cross-sectional study*	China 2018	Estudo transversal
9^(20)^	*Predictors of work-related stress among nurses working in primary and secondary health care levels in Dammam, Eastern Saudi Arabia*	Arábia Saudita 2014	Estudo transversal
10^(21)^	*Prevalence and associated factors of stress among primary health care nurses in Saudi Arabia, a multi-center study*	Arábia Saudita 2021	Estudo transversal
11^(22)^	*Stress and turnover intention among healthcare workers in Saudi Arabia during the time of COVID-19: Can social support play a role?*	Arábia Saudita 2021	Estudo transversal
12^(23)^	*The association of primary healthcare nurses’ perceived stress with organizational culture and climate in a team context*	Lituânia 2019	Estudo transversal
13^(24)^	*Job stress among general practitioners and nurses in primary care in England*	Inglaterra 1999	Estudo descritivo

**Quadro 2:** Abordagem do estresse

Abordagem do estresse		
Código ID	Caracterização do estresse	Aplicabilidade dos estudos
1^(12)^	A sobrecarga de trabalho, déficit de qualificação profissional, conflitos interpessoais, idade, sexo, estado civil e nacionalidade constituíram os fatores que contribuíram para o estresse.	Os programas de educação continuada para enfrentamento do estresse das enfermeiras devem ser formulados de acordo com o tipo de local de trabalho. As políticas públicas devem se centrarno estabelecimento de um ambiente de trabalho saudável, livre de estresse, de escassez de pessoal e de uma elevada carga de trabalho.
2^(13)^	O estresse esteve relacionado à elevada demanda psicológica e alto grau de exigência profissional.	Os reflexos negativos na saúde mental do trabalhador precisam ser mitigados por meio de estratégias de promoção da saúde, qualidade de vida e apoio social no ambiente de trabalho da Atenção Primária à Saúde.
3^(14)^	Considerou aspectos psicossociais relacionados ao trabalho, com base no modelo de demanda-controle e apoio social postulado por Karasek e Theorell.	Os empregadores podem investir na melhoria das condições de trabalho, e os órgãos a nível nacional podem enfatizar a fiscalização e supervisão, a fim de reduzir o percentual de trabalhadores acometidos pelas doenças causadas pela tensão mental.
4^(15)^	Abordagem do estresse percebido e aspectos individuais, profissionais e de grupo, incluindo os riscos psicossociais e ambientais relacionados ao trabalho.	O estudo foca em ações com bom custo-efetividade para propiciar melhores condições de saúde do trabalhador voltadas a minimizar problemas relacionados ao trabalho que possam causar um adoecimento, bem como promoção saúde física, mental e social.
5^(16)^	Abordagem do estresse organizacional.	A necessidade de organização do trabalho, promoção da educação continuada, melhora estrutural e planejamento são elementos essenciais para minimizar os estressores do âmbito organizacional do trabalho.
6^(17)^	A sobrecarga, escassez de recursos e incompletude da equipe são fatores que contribuem para o estresse dos trabalhadores da atenção primária.	Estratégias eficazes por parte dos sistemas de saúde são fundamentais, e devem ser adotadas visando ao investimento em profissionais, recursos adequados para a ampliação dos serviços e redução da burocracia administrativa.
7^(18)^	O estresse é abordado a partir das concepções dos sujeitos sobre o que causa o estresse.	O estudo aborda a necessidade contínua de desenvolver uma compreensão sólida das principais causas do estresse no trabalho e das suas inter-relações, uma vez que as consequências de uma força de trabalho de enfermagem estressada e desmoralizada interferem na qualidade do atendimento ao paciente e no perfil de risco.
8^(19)^	O estresse se relaciona à percepção que o indivíduo tem das exigências que lhes são feitas e da sua capacidade para satisfazer essas exigências.	Os gestores devem intervir por meio de programas que favorecem ações de educação em serviço na perspectiva de adotar medidas de alívio do estresse visando ao equilíbrio entre trabalho e família, redução de turnos de trabalho, e apoio ao desenvolvimento de carreira, salários e benefícios.
9^(20)^	O estresse relacionado ao trabalho é causado ou agravado pelo trabalho. É uma preocupação substancial e crescente para os trabalhadores, defensores, empregadores, reguladores de saúde e segurança ocupacional, e programas de compensação trabalhistas.	Recomenda-se uma estratégia apropriada na organização de cuidados de saúde para investigar a gestão do estresse em ambientes de cuidados de saúde. Além disso, devem ser desenvolvidos programas de intervenção para identificar e aliviar as fontes e os efeitos do estresse, incluindo mais formação, apoio e melhores condições de trabalho.
10^(21)^	O estresse ocupacional foi classificado como sentimento percebido negativamente, devido à incapacidade de lidar com demandas organizacionais do trabalho.	É necessária uma abordagem interventiva abrangente para minimizar e prevenir o estresse entre as enfermeiras. As intervenções organizacionais devem ser destinadas à redução do estresse e a mitigar o impacto dos fatores de estresse no local de trabalho, incluindo carga de trabalho e programa de treinamento em gerenciamento de estresse.
11^(22)^	Apontou o apoio social como estratégia para minimizar o impacto do estresse.	O apoio social pode ser usado como uma estratégia de enfrentamento para reduzir o estresse e a rotatividade entre os profissionais de saúde.
12^(23)^	A qualidade do atendimento ao usuário, má comunicação e falta de apoio foram apontadas como fatores que impedem as enfermeiras de utilizar o seu potencial para prestar cuidados da mais alta qualidade aos seus pacientes, e isso favorece o estresse.	Recomenda-se a realização de novos estudos para estabelecer o impacto da relação entre cultura e clima organizacional no estresse.
13^(24)^	Considerou a satisfação no trabalho e bem-estar mental para abordagem do estresse.	Tem havido pouco trabalho sobre os efeitos da saúde mental em relação aos cuidados aos pacientes e às suas consequências, o que reforça que esta é uma área que requer mais pesquisas.

Para a caracterização dos estudos, foram considerados o código de identificação do artigo, país/ano e tipo de estudo, conforme apresentado no Quadro 1.

Para a abordagem do estresse, foram considerados aspectos relacionados ao artigo por meio do código de identificação, caracterização do estresse e aplicabilidade dos estudos (Quadro 2).

## DISCUSSÃO

De acordo com os estudos que compõem a amostra, apresentados no Quadro 1, verificou-se que os títulos apresentados estão relacionados ao estresse no trabalho no contexto da APS. O período de publicação foi de 1999 a 2023, sendo quatro na Arábia Saudita, dois, na Lituânia, um, no Brasil, um, no Chile, um, na Bósnia e Herzegovina, um, nos Estados Unidos, um, no Reino Unido, um, na China, e um, na Inglaterra. Quanto ao tipo de estudo, identificaram-se oito estudos transversais, três estudos descritivos, um estudo exploratório e um estudo misto (qualitativo e quantitativo)^(12-24)^.

Com relação às revisões encontradas, as mesmas abordavam o estresse em termos gerais^(25-48)^. Houve dificuldade de encontrar revisões que avaliassem apenas o estresse das enfermeiras no trabalho na APS. Portanto, houve a preocupação de selecionar os estudos considerando a semelhança entre os serviços de saúde e a atuação da enfermeira, constatando-se que o estresse está presente no público-alvo investigado.

Nessa perspectiva, artigo de revisão integrativa apresentou que a prevalência de estresse em trabalhadoras na APS foi classificada de baixa a moderada, estando associado aos conflitos entre membros da equipe, principalmente médicas e enfermeiras. Além disso, ressalta-se que as diversas interfaces da jornada de trabalho no ambiente laboral e domiciliar, bem como o cuidado com a família, tornam a enfermeira mais propensa ao estresse^(43)^.

Com relação à abordagem do estresse (Quadro 2), o mesmo foi caracterizado pela sobrecarga de trabalho, déficit de qualificação profissional e conflitos interpessoais, incluindo aspectos sociodemográficos que favorecem o estresse psicológico, como idade, sexo, estado civil e nacionalidade. Ademais, o estresse esteve relacionado à elevada demanda psicológica, ao alto grau de exigência profissional e à falta de apoio social^(12-24)^.

De forma complementar, as evidências indicam que o baixo apoio social no ambiente de trabalho está associado a aspectos sociais e psicológicos, como o estresse ocupacional. Por outro lado, o elevado apoio social contribui para a proteção contra os efeitos deletérios à saúde mental, favorecendo a capacidade laboral. Dessa forma, é recomendado que sejam realizadas intervenções e implementação do apoio social de forma humanizada, segura e com reconhecimento profissional, com enfoque na redução da sobrecarga de trabalho e dos efeitos nocivos do ambiente de trabalho^(44)^.

Quanto à aplicabilidade dos estudos (Quadro 2), entre as medidas apontadas para minimizar os impactos do estresse no trabalho, destacaram-se os programas de educação continuada direcionados ao local de trabalho e as políticas públicas de saúde do trabalhador no contexto da APS como estratégias para se ter um espaço de trabalho seguro, acolhedor, livre de estresse, da escassez de pessoal e de uma elevada carga de trabalho^(12-24)^.

Entretanto, foi enfatizada a carência do desenvolvimento de ações visando à promoção da saúde, qualidade de vida e apoio social. Investimentos na melhoria das condições de trabalho, bem como fiscalização e supervisão por parte dos órgãos nacionais, foram apresentados como medidas que podem prevenir ou reduzir o surgimento de doenças ou agravos ocupacionais. Quanto à estrutura organizacional e ao planejamento, quando bem estruturados, apresentaram-se como elementos essenciais para minimizar os riscos e, consequentemente, o estresse psicológico^(12-24)^.

Tais achados demonstram que, no âmbito da APS, são imprescindíveis o cuidado e o monitoramento da saúde física e psíquica das enfermeiras, considerando os estressores e os riscos ocupacionais existentes no ambiente laboral. A reorganização do processo de trabalho, o uso das tecnologias em saúde de maneira adequada e a adoção de medidas preventivas, como melhoria das condições de trabalho, gestão de conflitos, atividades de educação permanente em equipe, pausas na jornada de trabalho, valorização profissional, desenvolvimento de políticas de humanização, acompanhamento psicológico e suporte para o estresse ocupacional, são estratégias que devem ser adotadas com o objetivo de tornar a jornada de trabalho menos exaustiva e mais produtiva, com menos estresse e adoecimento^(45)^.

Diante disso, os estudos analisados mostraram ainda que melhores condições de trabalho requerem intervenções organizacionais e individuais, envolvendo o investimento em profissionais, aplicação adequada de recursos para ampliação dos serviços, redução da burocracia administrativa, adoção de medidas de alívio do estresse, redução dos turnos de trabalho, redução da rotatividade de profissionais, apoio ao desenvolvimento de carreira, salários e benefícios, treinamento de gerenciamento do estresse, e programas de enfrentamento^(12-24)^.

Em consonância com os achados, estudo de revisão reforçou que a precarização das condições de trabalho das enfermeiras é agravada pela escassez de recursos materiais, déficit de profissionais, vínculos empregatícios frágeis e baixos salários. Esse cenário contribui para a sobrecarga das profissionais, que também estão expostas a risco físicos, químicos, biológicos e ergonômicos, tornando-se vulneráveis ao desenvolvimento de doenças físicas e mentais, interferindo na qualidade de vida e na assistência prestada. A competência exigida às enfermeiras e o desequilíbrio entre as demandas e as condições de trabalho provocam exaustão, estresse, danos psicológicos e fisiológicos^(46)^.

No entanto, constatou-se que a constituição de ambientes de trabalho saudáveis na APS, livre do estresse e dos estressores, configura um desafio para gestores e trabalhadores que vislumbram estratégias para o enfrentamento das diversas situações que limitam e, por vezes, inviabilizam a adoção de medidas de proteção individual e coletiva. Portanto, o processo de modificação da realidade é complexo, exigindo mobilização e diálogo entre os atores, e luta para consolidação de políticas públicas de saúde do trabalhador^(48)^.

Corroborando a temática, artigo de revisão integrativa apontou que a qualificação de trabalhadores da APS é um componente essencial para a melhoria da assistência prestada na saúde^(47)^. Sendo assim, torna-se fundamental compreender e acolher as demandas de atualização e aperfeiçoamento profissional, já que a APS tem como proposta orientar políticas de fortalecimento dos sistemas de saúde, acolhendo os seus usuários de forma integral, eficaz e eficiente.

### Limitações do estudo

As limitações da presente revisão de escopo abrangem a escassez de estudos que abordem o estresse das enfermeiras atuantes na APS, especialmente no Brasil. Esse contexto demanda mais estudos que explorem a temática e contribuam para preencher as lacunas existentes na literatura.

Além disso, a heterogeneidade dos estudos compromete parcialmente a resposta direta à pergunta de pesquisa, podendo interferir na interpretação e generalização dos achados da revisão.

### Contribuições para a área da enfermagem, saúde ou políticas públicas

Esta revisão de escopo permitiu identificar a origem do estresse das enfermeiras da APS, mostrando que se faz urgente a reorganização dos serviços da APS, perpassando a reforma orçamentária da saúde, para que o planejamento em saúde seja exequível, com melhores condições de trabalho, valorização profissional, autonomia e respeito.

Por meio desses resultados, com a identificação dos fatores contribuintes para o estresse, sugere-se que medidas possam ser adotadas pelos profissionais em loco como: dividir tarefas e atribuições com todos os membros da equipe; realizar o reconhecimento da área de atuação; promover reunião com líderes comunitários e representantes da população adscrita para conhecer as suas necessidades e, assim, planejar e implementar ações para atendê-los, direcionando, de maneira mais eficiente e eficaz, os recursos disponíveis; e oferecer apoio social e psicológico para as enfermeiras da APS.

## CONCLUSÕES

Os estudos sinalizaram que o estresse psicológico apresentado pelas enfermeiras da APS decorre da exposição aos estressores existentes no ambiente laboral e as principais causas estavam relacionadas às condições de trabalho, a organização e a gestão. Assim, o estresse psicológico, caracterizou-se pelas más condições e sobrecarga de trabalho, déficit na qualificação profissional, conflitos interpessoais, elevada demanda psicológica, alto grau de exigência profissional, falta de apoio social, bem como de recursos humanos, financeiros e materiais.

Constatou-se também a influência de fatores individuais e profissionais, de questões familiares, da capacidade de atender às exigências do serviço e de lidar com as demandas organizacionais, todos eles estressores presentes no ambiente laboral.

Recomenda-se atentar para a necessidade de reorganização do trabalho das enfermeiras da APS, visando à qualificação profissional, à melhora das condições de trabalho, à identificação dos estressores, à eliminação dos riscos ocupacionais, à infraestrutura adequada, e aos recursos humanos, materiais e financeiros suficientes para atender às demandas.

Diante do exposto, os estudos enfatizaram medidas que visam prevenir ou reduzir o estresse psicológico das enfermeiras na APS, incluindo os programas de educação continuada um ambiente de trabalho saudável com vistas à promoção da saúde, qualidade de vida e bem-estar.

## Data Availability

Os dados de pesquisa estão disponíveis no corpo do artigo.
